# Linking organismal growth, coping styles, stress reactivity, and metabolism via responses against a selective serotonin reuptake inhibitor in an insect

**DOI:** 10.1038/s41598-018-26722-9

**Published:** 2018-06-05

**Authors:** Indrikis Krams, Giedrius Trakimas, Sanita Kecko, Didzis Elferts, Ronalds Krams, Severi Luoto, Markus J. Rantala, Marika Mänd, Aare Kuusik, Jukka Kekäläinen, Priit Jõers, Raine Kortet, Tatjana Krama

**Affiliations:** 1 Institute of Ecology and Earth Sciences, University of Tartu, Tartu, Estonia; 2 Department of Zoology and Animal Ecology, Faculty of Biology, University of Latvia, Rīga, Latvia; 3 University of Tennessee, Department of Psychology, Knoxville, USA; 4 Institute of Biosciences, Vilnius University, Vilnius, Lithuania; 5 Department of Biotechnology, Institute of Life Sciences and Technology, Daugavpils University, Daugavpils, Latvia; 6 Department of Botany and Ecology, Faculty of Biology, University of Latvia, Rīga, Latvia; 7 English, Drama and Writing Studies, University of Auckland, Auckland, New Zealand; 8 School of Psychology, University of Auckland, Auckland, New Zealand; 9 Department of Biology & Turku Brain and Mind Centre, University of Turku, Turku, Finland; 10 Department of Plant Protection, Institute of Agricultural and Environmental Sciences, Estonian University of Life Science, Tartu, Estonia; 11 Department of Environmental and Biological Sciences, University of Eastern Finland, Joensuu, Finland; 12 Insttute of Molecular and Cell Biology, University of Tartu, Tartu, Estonia

## Abstract

Evidence suggests that brain serotonin (5-HT) is one of the central mediators of different types of animal personality. We tested this assumption in field crickets *Gryllus integer* using a selective serotonin reuptake inhibitor (SSRI). Crickets were selected for slow and rapid development and tested for their coping styles under non-stressful conditions (time spent exploring a novel object). Resting metabolic rate, maximum metabolic rate and latency to resume activity were measured under stressful conditions (stress reactivity). Measurements were taken (i) before and (ii) during the SSRI treatment. Before the SSRI treatment, a strong negative correlation was observed between coping style and stress reactivity, which suggests the existence of a behavioral syndrome. After the SSRI treatment, the syndrome was no longer evident. The results of this study show that 5-HT may be involved in regulating behavior not only along a stress reactivity gradient but also along a coping styles axis. The relationship between personality and the strength and direction of 5-HT treatment on observed behaviors indicates trait-like individual differences in 5-HT signaling. Overall, these findings do not support recent ideas arising from the pace-of-life syndrome (POLS) hypothesis, which predict higher exploration and metabolic rates in rapidly developing bold animals.

## Introduction

Phenotypic differences in trait expression are a prerequisite for evolution through natural selection. Although most of the traits of an organism vary around a mean value, research on human and animal behavior has revealed between-individual clusters in behavioral responses, often attributed to different personality axes that are called *personality types*, *behavioral predispositions*, *temperaments* or *coping styles*. Some of these behavioral responses are not heritable^1,2^, while others demonstrate a negligible or a considerable degree of heritability^3–7^. Animal personality is tightly linked to individual life histories, survival and fitness^5,6,8–13^.

The pace-of-life syndrome (POLS) hypothesis, rooted in the classic concept of *r*- and *K*-selection, is particularly relevant in this context^14–16^, as it suggests that life history traits such as age at maturity and growth rate are likely coupled with individually consistent behaviors and immune responses. For example, shy individuals often grow slower, reach larger body size, mature later, invest more in immune system and have longer hiding time in an anti-predator context under familiar conditions than bold individuals^16^. Aggressiveness of bold individuals hypothetically facilitates acquiring and monopolizing resources and may be linked to a fast growth rate and more intense reproductive effort early in life^5,17^.

Personality research is a complex field that adjoins behavioral ecology with psychology and molecular biology. It is therefore important to define key terms before testable hypotheses are proposed. The term *behavioral syndrome* indicates that behavioral trait characteristics involve suites of correlated behaviors^18,19^ that are consistent over time and across contexts. The term *coping style* is more commonly used to characterize individual trait differences in biomedicine, and this concept has been useful in explaining behavioral responses and their underlying physiological mechanisms. The coping style concept implies that animals may react to a stimulus with alternative response patterns. According to the two-tier model of animal personality proposed by Steimer *et al*.^20^, coping styles reflect the quality of the response to a stressor, while stress reactivity reflects the quantity of the response expressed. In this model, *bold* individuals are those with a strong tendency to act (proactivity) combined with low stress reactivity. *Docile* individuals can be characterized as a combination of reactive coping associated with low stress reactivity. The combination between high stress reactivity and a high tendency for proactive coping is labeled as *panicky*. *Shy* individuals exhibit reactive coping style and high stress reactivity.

Although those personality characteristics are common, researchers rarely use all of the above terms because that would require the use of several tests for each individual being studied. For example, stress reactivity needs to be measured by obtaining levels of stress hormones/neuromediator concentrations, or by observing the organisms under stressful conditions. In contrast, reactive and proactive coping styles differ in behavioral flexibility, where the proactive individuals act on the basis of previous experiences and develop routines in stable environments^21,22^. The reactively coping animal tends to rely more on a detailed appraisal of their current environment because they react to immediate environmental stimuli and tend to explore any changes in their environment.

The metabolic rate of an organism varies with its body size and body temperature^23,24^. The POLS hypothesis proposes that rapidly developing, often bold individuals have life histories involving high daily energy expenditures and higher basal or resting metabolic rates (RMR) because they are forced to process more food and inevitably excrete more waste products at a faster rate compared to slowly developing individuals of same size. Rapidly developing individuals may, however, remain smaller than slow developers^16^. Thus, personality also has the potential to affect the metabolic rate of an organism. Another important but often neglected source of variability in RMR arises because individuals consistently differ in their stress responses to conditions in which metabolism measurements are performed^25,26^. For example, because reactive individuals (shy, docile) often become immobile under stressful conditions^12,13^, their metabolic rates may be mistakenly classified as RMR^27^. Recent research shows that while the latency to resume body movements after handling in a familiar environment is longer for slowly developing crickets, they resume body movements sooner than rapidly developing crickets when in a more stressful, unfamiliar environment^28^. Slowly developing crickets were found to have significantly higher RMR compared to rapidly developing crickets. It is important to note that according to established experimental procedure, measurements of respiration always require placing an organism in a novel environment^27^. This has the potential to induce significant stress, which may force less stress-resistant animals to stay active during respirometry to explore or escape their respirometry chambers. This demonstrates the importance of finding the proper approach to measurements of stress reactivity and metabolism, as well as making a correct interpretation of the results obtained to explain the relationships between animal personality and underlying physiological mechanisms^29^.

Behavioral responses of shy, often anxious individuals are associated with high concentrations of stress hormones^30^, implying that their slowness and caution are caused by physiological stress. In psychology and physiology, shyness is usually defined as the feeling of anxiety and awkwardness when an individual is under conditions of stress. Stronger forms of shyness are usually referred to as *anxiety* and elevated *neuroticism*. In extreme cases, it might be related to a depression-like psychological condition, which includes the experience of fear to the extent of inducing panic^31^. This calls for reliable tests to discriminate between shy and panicky personality types^20,29^.

In the central nervous system, serotonin (5-hydroxytryptamine: 5-HT), a monoamine neurotransmitter^32^, has several important functions, including the regulation of aggression, mood, sleep, dispersal and metabolic rate^33–36^. Modulation of serotonin at synapses of the brain is thought to be the key mechanism of several classes of pharmacological antidepressants. Experimental evidence has revealed links between low levels of 5-HT and the development of anxiety and stress-prone behavioral phenotypes^37,38^. However, an association between low concentrations of 5-HT and proactive coping style^39^ suggests that 5-HT may be involved in the regulation of behavior not only along the stress reactivity gradient but also along the coping styles axis^20,29,40^ (but see^34^). These findings indicate that 5-HT potentially affects personality types in a more complex way, which should be taken into account when testing the influence of pharmacological compounds on behavioral responses under stress.

Field crickets are a suitable and often-used model group for animal personality research^41–43^. In this study, we compared coping style differences between slowly and rapidly developing western stutter-trilling crickets (*Gryllus integer*) under non-stressful and stressful conditions. Prior to the study, the crickets had been selected for slow and rapid developmental time over five generations. We also measured their RMR, maximum metabolic rate (MMR) and the latency to resume activity under stressful conditions in the insect chamber of respirometer. This was done twice: before a selective serotonin reuptake inhibitor (SSRI) was added to the food and once again during the anti-depressant treatment.

We expected greater effects of anti-depressant treatment on behavioral responses of proactive individuals (bold, panicky) − those paying little attention to minor changes in their environment. Although some earlier evidence suggested that central injection of 5-HT or its precursor 5-hydroxytryptophan (5-HTP) may cause increased metabolic rates^44,45^ (but see^46^), other research has shown that serotonin deficiency significantly increases amino acid, energy, purine, lipid and gut microflora metabolisms and rates of oxidative stress^47–49^. Therefore, we predicted an SSRI-induced decrease in the metabolic rate of crickets with high stress reactivity (shy, panicky), especially in panicky individuals^50,51^, because 5-HT is likely to influence proactive individuals (panicky, bold) more than reactive ones (shy, docile)^36,52^.

## Results

### Body mass

**Figure 1:**
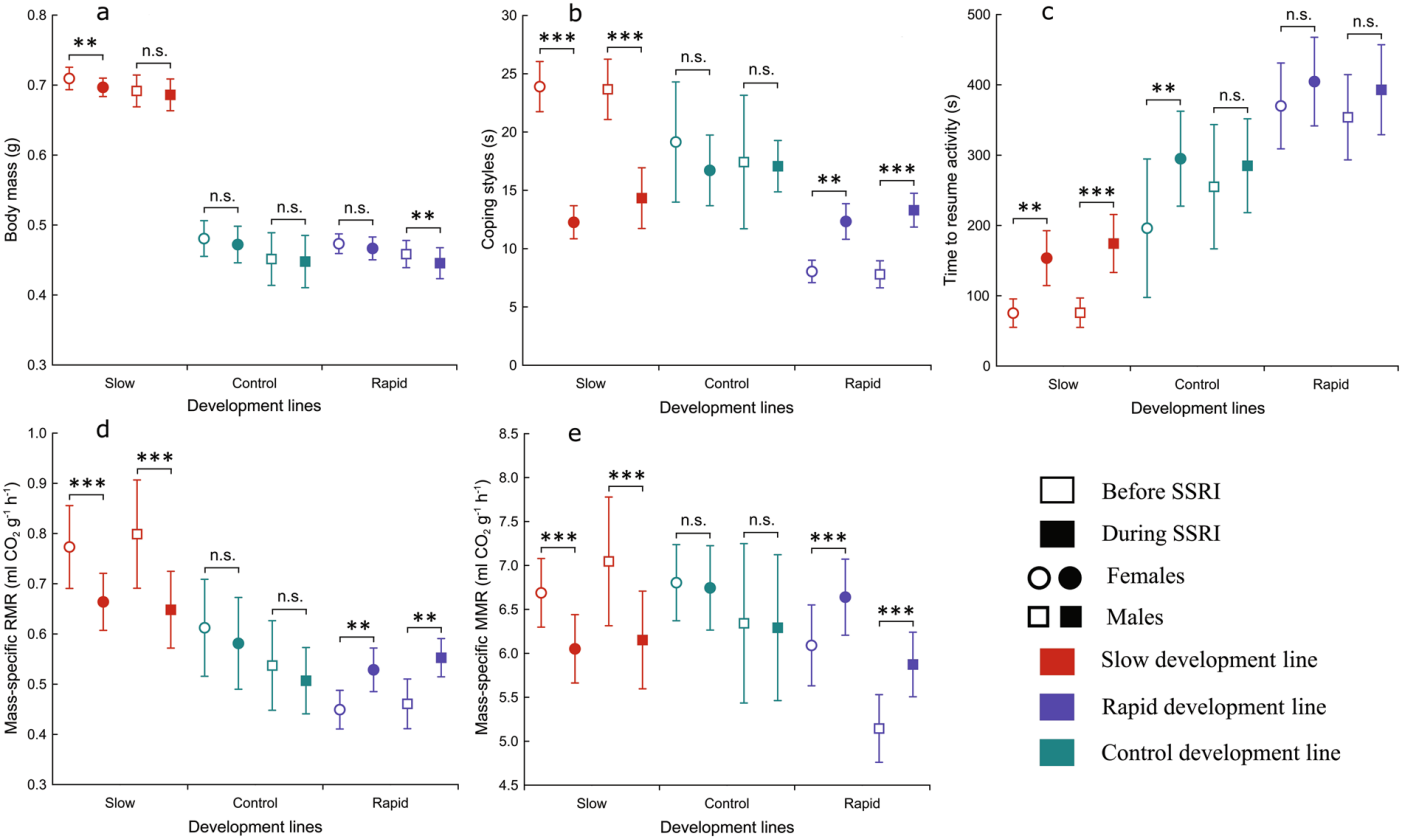
Mean (±95%CI) body mass (**a**), coping styles (**b**), time to resume activity (**c**), RMR (**d**) and MMR (**e**) before SSRI (▫, ◦) and after SSRI treatment (▪, ⦁) in crickets of rapid (n = 41), slow (n = 37) and control (n = 28) developmental lines. ◦, ⦁ indicate females, ▫, ▪ indicate males.

**Figure 2:**
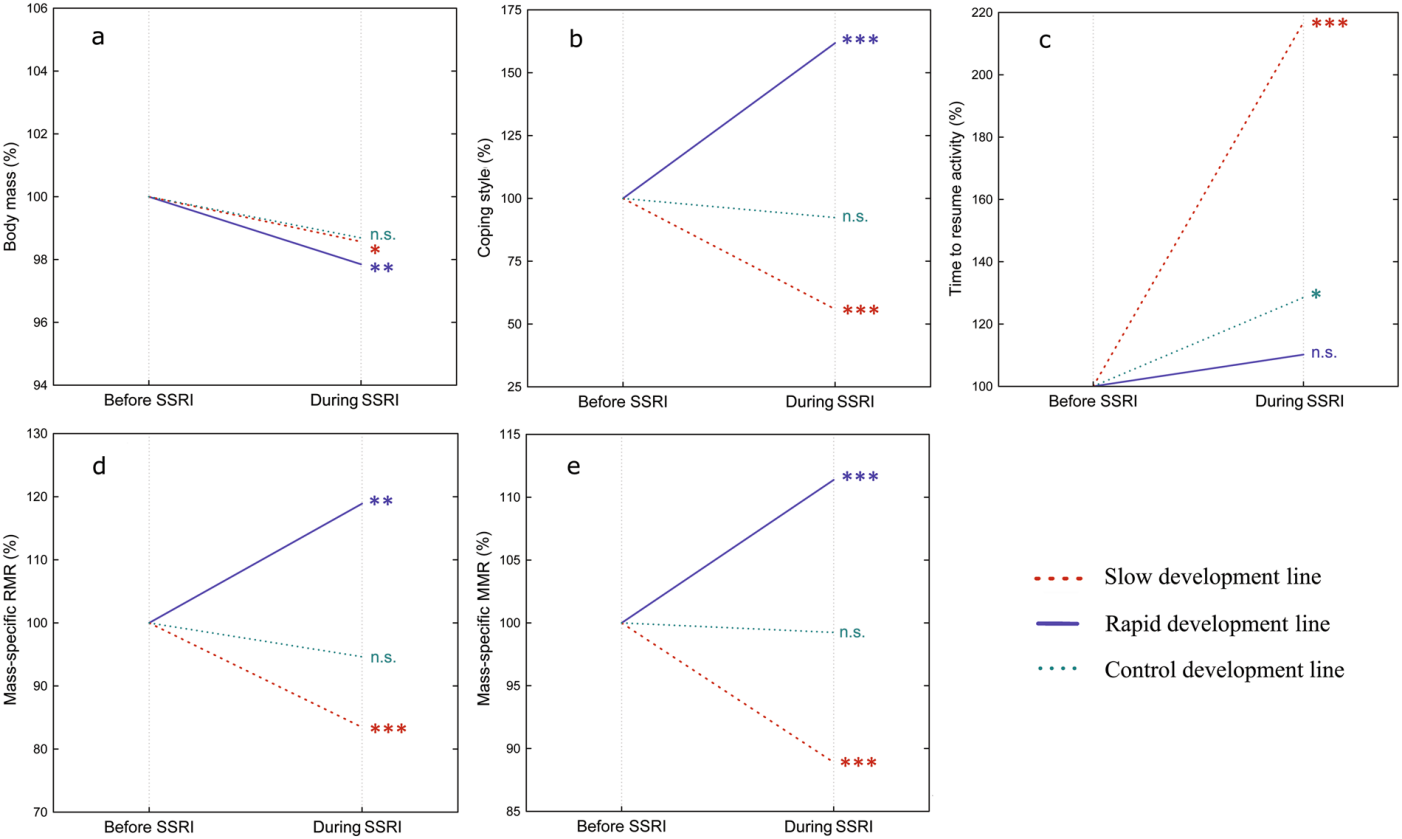
Relative change in mean body mass (**a**), coping styles (**b**), time to resume activity (**c**), RMR (**d**) and MMR (**e**) before SSRI and after SSRI treatment in crickets of rapid, slow and control developmental lines. Significance based on Tukey’s test: n.s. *P* > 0.05, *P < 0.05, **P < 0.01, ***P < 0.001.

Our results demonstrate that SSRI can have relatively small but significant effects on body mass of crickets (rmANOVA, *F*_(1, 100)_ = 22.27, *P* < 0.0001, partial η^2^ = 0.18, Figs 1a and 2a). The average body mass of crickets decreased from 0.548 ± 0.004 g to 0.539 ± 0.004 g (unweighted means ± SE) during SSRI treatment. The body mass of crickets largely depended on the developmental line (rmANOVA, *F*_(2, 100)_ = 356.9, *P* < 0.0001, partial η^2^ = 0.85). The effect of sex, although much smaller than that of developmental line, was significant (rmANOVA, *F*_(1, 100)_ = 5.41, *P* = 0.02, partial η^2^ = 0.05). Before the SSRI treatment, slowly developing crickets were heavier (0.701 ± 0.006 g (weighted means ± SE) than rapidly developing (0.466 ± 0.007 g) and control crickets (0.466 ± 0.011 g), while females (0.558 ± 0.016 g) were heavier than males (0.537 ± 0.017 g). Although body mass decreased in males and females of all developmental lines during the SSRI treatment, slowly developing crickets remained the heaviest among the lines, while females were heavier than males. All possible interactions between factors were non-significant. No aging and testing apparatus effects were evident (rmANOVA, *F*_(1, 54)_ = 1.0, *P* = 0.323, partial η^2^ = 0.02). Body mass was repeatable in control (*R* = 0.69) and highly repeatable in slowly (*R* = 0.93) and rapidly developing crickets (*R* = 0.94; Table 1).

**Table 1:** Repeatabilities of traits in crickets of rapid (n = 41), slow (n = 37) and control (n = 28) developmental lines estimated using two-way random effects model for single measures.

Variables	Control			Slow			Rapid		
	R	95%CI limits	F_19,19_	R	95%CI limits	F_19,19_	R	95%CI limits	F_19,19_
Body mass	0.69	0.38; 0.86	5.75^***^	0.93	0.82; 0.97	25.1^****^	0.94	0.85; 0.98	29.8^****^
Coping styles^a^	0.91	0.80; 0.97	21.4^****^	0.67	0.33; 0.85	4.95^***^	0.16	−0.15; 0.50	1.58^ns^
Stress reactivity	0.99	0.98; 1.00	226.3^****^	0.92	0.82; 0.97	25.5^****^	0.97	0.93; 0.99	66.4^****^
RMR	0.96	0.90; 0.98	51.1^****^	0.91	0.80; 0.97	22.9^****^	0.79	0.51; 0.92	10.2^****^
MMR	0.94	0.87; 0.98	33.7^****^	0.94	0.86; 0.98	33.5^****^	0.94	0.86; 0.98	32.4^****^

^***^P < 0.001; ^****^P < 0.0001; ^ns^ *P* > 0.05; ^a^combined all selection lines: R = 0.94 (0.91; 0.97), F_59,59_ = 33.8, *P* < 0.001.

### Coping style

**Figure 3:**
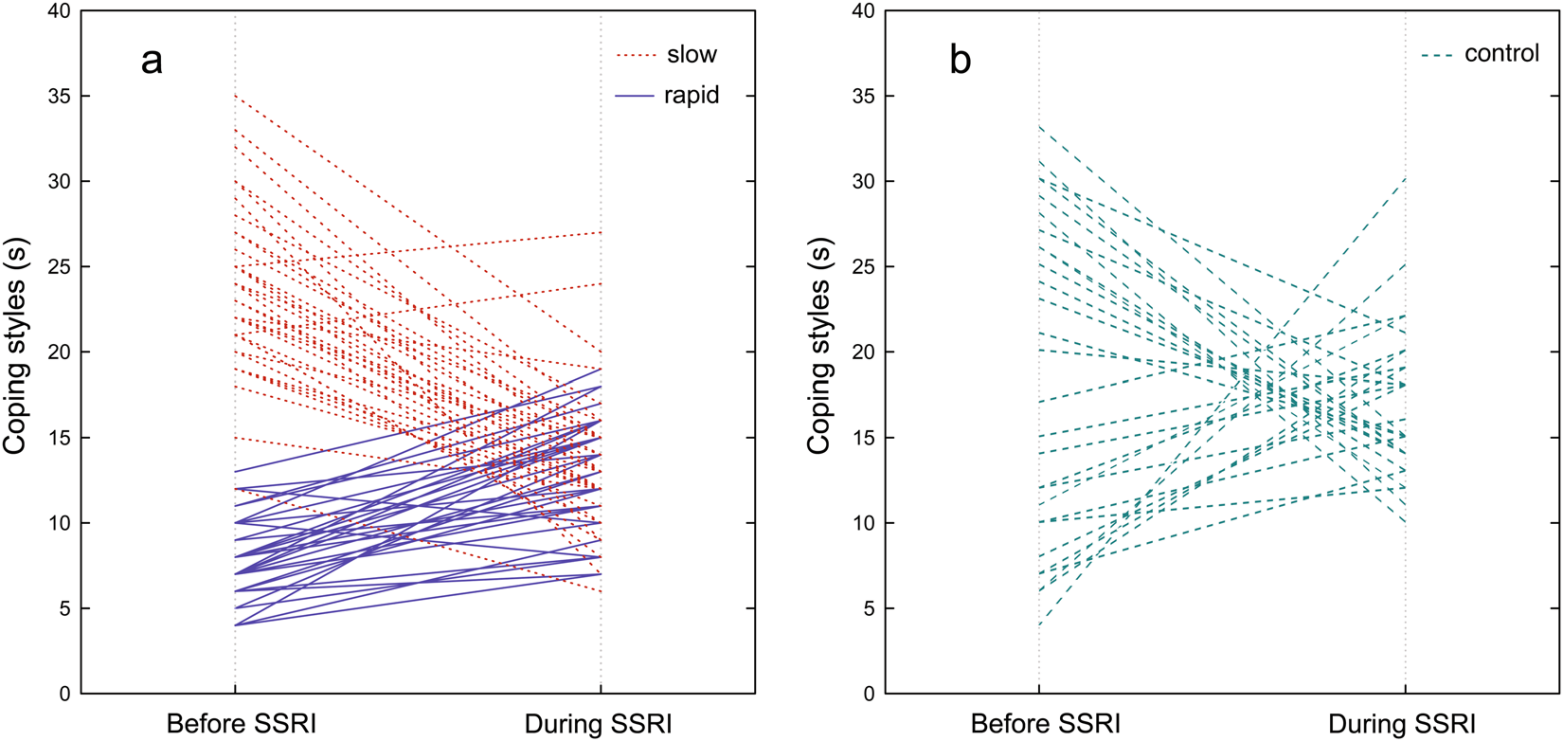
Change in coping styles after SSRI treatment in individual crickets of rapid (──, n = 41) and slow (……, n = 37) developmental lines (**a**) and control (n = 28) line (**b**).

We found significant main effects of developmental lines (rmANOVA, *F*_(2, 100)_ = 70.45, *P* < 0.0001, partial η^2^ = 0.56) and SSRI treatment (rmANOVA, *F*_(1, 100)_ = 10.19, *P* = 0.002, partial η^2^ = 0.09) to coping styles of the crickets, while sex apparently had no effect on it (rmANOVA, *F*_(1, 100)_ = 0.1, *P* = 0.76, partial η^2^ = 0.001) (Figs 1b and 2b). However, there was significant interaction between developmental line and antidepressant treatment (*F*_(2, 100)_ = 42.03, *P* < 0.0001, partial η^2^ = 0.46), suggesting that simultaneous effects of those factors on the coping style were not additive. Before the SSRI treatment, slowly developing crickets explored the novel object longer than control (Bonferroni test: *P* = 0.0002) and rapidly developing crickets (Bonferroni test: *P* < 0.0001); control crickets explored the object longer than rapidly developing individuals (Bonferroni test: *P* < 0.0001). During the SSRI treatment, rapidly developing crickets decreased their proactivity by increasing exploration time of the novel object (Bonferroni test: *P* = 0.0008). Slowly developing crickets decreased the proportion of time devoted to exploration (Bonferroni test: *P* < 0.0001) (Fig. 3), while the control individuals explored the novel object equally long (Bonferroni test: *P* > 0.05). After the SSRI treatment, coping styles of all developmental lines became statistically similar (Bonferroni test: *P* > 0.05). No aging nor testing apparatus effects were evident (rmANOVA, *F*_(1, 54)_ = 0.93, *P* = 0.339, partial η^2^ = 0.02). Coping styles were repeatable in the control (*R* = 0.91) and slowly developing crickets (*R* = 0.67) but not in the rapidly developing crickets (*R* = 0.16; Table 1). However, combined repeatability was high (*R* = 0.94) when calculated for all selection lines together.

### Stress reactivity

We found significant main effects of antidepressant treatment on time to resume activity in the insect chamber (rmANOVA, *F*_(1, 100)_ = 33.83, *P* < 0.0001, partial η^2^ = 0.25) (Figs 1c and 2c). Time to resume activity increased from 221 ± 12 s (unweighted means ± SE) before the SSRI treatment to 284 ± 11 s after the treatment. Stress reactivity of the crickets depended on developmental line (rmANOVA, *F*_(2, 100)_ = 61.73, *P* < 0.0001, partial η^2^ = 0.55), while sex had no effect on stress reactivity (rmANOVA, *F*_(1, 100)_ = 0.12, *P* = 0.73, partial η^2^ = 0.001) either before or after the SSRI treatment. After adding antidepressants to the food, all selection lines increased their time to resume active movements in the insect chamber. No significant interactions were observed. No aging and testing apparatus effects were found (rmANOVA, *F*_(1, 54)_ = 0.41, *P* = 0.523, partial η^2^ = 0.01). Stress reactivity was highly repeatable in all selection lines (range *R* = 0.92–0.99; Table 1).

### Resting metabolic rate

**Figure 4:**
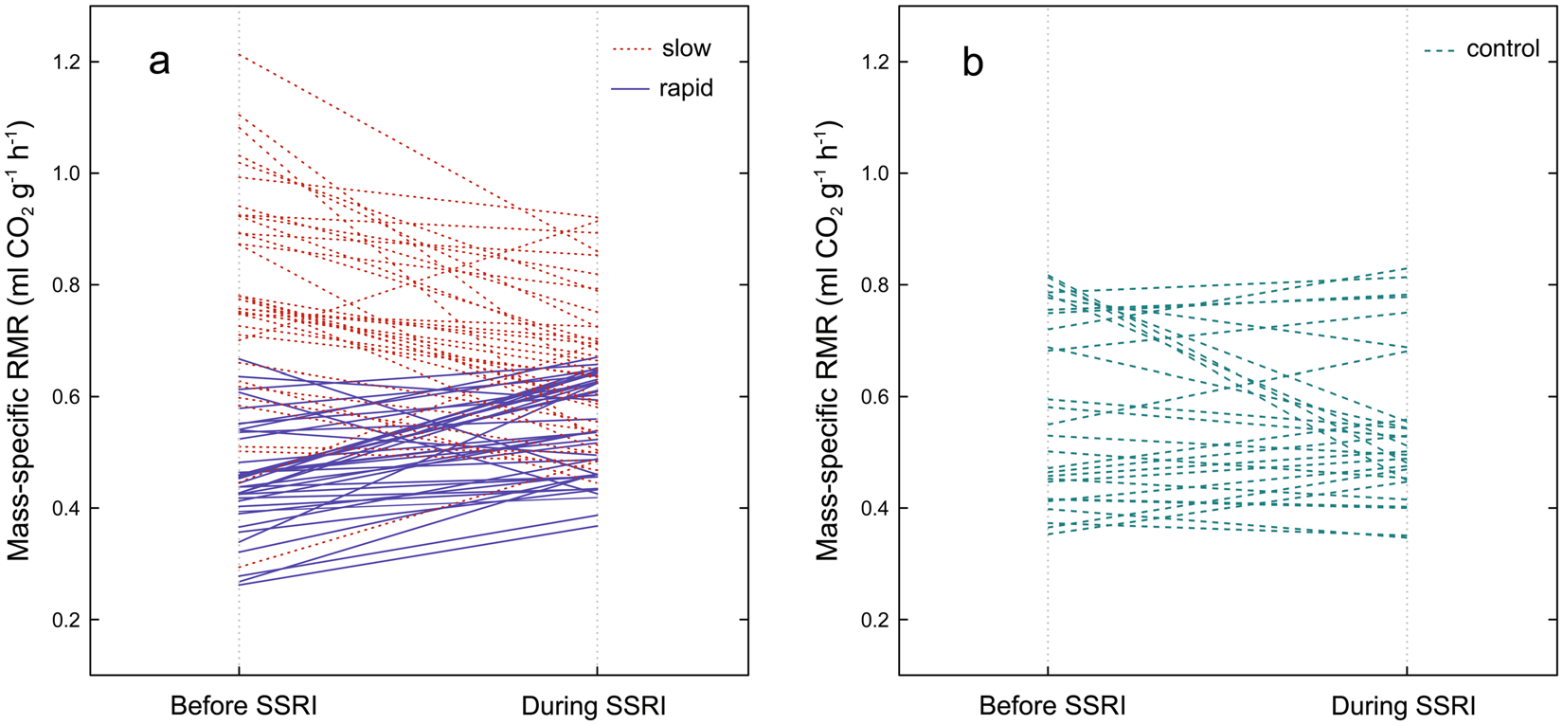
Change in RMR after SSRI treatment in individual crickets of rapid (──, n = 41) and slow (……, n = 37) developmental lines (**a**) and control (n = 28) line (**b**).

A significant main effect of developmental line on RMR was observed (rmANOVA, *F*_(2, 100)_ = 34.63, *P* < 0.0001, partial η^2^ = 0.41) (Figs 1d and 2d). However, there was a significant interaction between developmental line and antidepressant treatment (rmANOVA, *F*_(2, 100)_ = 25.52, *P* < 0.0001, partial η^2^ = 0.38). Before the SSRI treatment, the RMR of slowly developing crickets was higher than that of control (Bonferroni test: *P* < 0.0001) and rapidly developing crickets (Bonferroni test: *P* < 0.0001). The RMR of rapidly developing crickets was lower than that of control crickets (Bonferroni test: *P* = 0.008). After the treatment, RMR significantly increased in rapidly developing crickets (*P* = 0.001), significantly decreased in slowly developing crickets (Bonferroni test: *P* < 0.0001) (Fig. 4), but remained the same in controls (Bonferroni test: *P* = 1). After the antidepressant treatment, the RMR of slowly developing crickets was statistically similar to the RMR in the control line before the SSRI treatment (Bonferroni test: *P* = 0.29). As the mean RMR data show, all the lines became more similar after the SSRI treatment. However, some of the developmental lines remained significantly different from each other (i.e., rapidly developing crickets *vs* slowly developing crickets: *P* = 0.004; slowly developing crickets *vs* control group: *P* = 0.02). RMR was repeatable in rapidly developing (*R* = 0.79), and highly repeatable in slowly developing (*R* = 0.91) and control crickets (*R* = 0.96; Table 1).

### Maximum metabolic rate

A small significant main effect of developmental line on MMR (rmANOVA, *F*_(2, 100)_ = 4.03, *P* = 0.02, partial η^2^ = 0.08) was observed, while sex had no effect on MMR (rmANOVA, *F*_(1, 100)_ = 2.26, *P* = 0.07, partial η^2^ = 0.03) (Figs 1e,2e). However, there was a significant interaction between developmental line and SSRI treatment (rmANOVA, *F*_(2, 100)_ = 64.38, *P* < 0.0001, partial η^2^ = 0.56). Before the SSRI treatment, slowly developing crickets had the highest rates of maximum metabolism, followed by control and rapidly developing crickets. Significant differences were found between rapidly and slowly developing lines (Bonferroni test: *P* < 0.0001) and between rapidly developing and control crickets (Bonferroni test: *P* = 0.006), while slowly developing crickets and the control group did not differ (Bonferroni test: *P* = 1). During the SSRI treatment, MMR significantly increased in rapidly developing crickets (Bonferroni test: *P* < 0.0001) and decreased in slowly developing individuals (Bonferroni test: *P* < 0.0001), while it did not change significantly in the control group (Bonferroni test: *P* = 1). After the SSRI treatment, the MMR of all developmental lines was statistically similar (Bonferroni test: *P* = 1). MMR was highly repeatable in all selection lines (all *R* = 0.94; Table 1).

### Associations among traits

**Figure 5:**
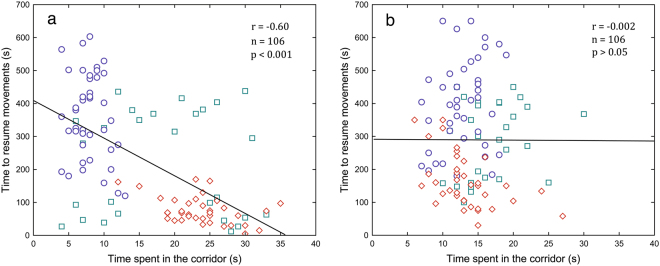
The two-tier model of cricket personality before the SSRI treatment (**a**), and after the SSRI treatment (**b**). ○ = rapid developmental line (n = 41), ◊ = slow developmental line (n = 37) and □ = control developmental line (n = 28).

Before the SSRI treatment, the crickets’ body mass was positively correlated with RMR (*r* = 0.63, *P* < 0.0001), MMR (*r* = 0.34, *P* < 0.01) and coping styles (*r* = 0.69, *P* < 0.0001), while being negatively associated with stress reactivity (*i.e*. time to resume activity) (*r* = −0.66, *P* < 0.0001, Table 2). After the SSRI treatment, the relationships between the crickets’ body mass, coping styles and MMR disappeared, while the associations were still apparent between body mass and stress reactivity (*r* = −0.63, *P* < 0.0001) and body mass and RMR (*r* = 0.40, *P* < 0.001). RMR before the SSRI treatment was positively related with MMR (*r* = 0.40, *P* < 0.001), coping styles (*r* = 0.69, *P* < 0.0001) and negatively with stress reactivity (*r* = −0.74, *P* < 0.0001). After the SSRI treatment, the relationships between RMR, coping style and stress reactivity were weaker (*r* = 0.33, *P* < 0.01; *r* = −0.32, *P* < 0.05, respectively, Table 2). Before the SSRI treatment, MMR was also associated with coping styles (*r* = 0.37, *P* < 0.01) and stress reactivity (*r* = −0.40, *P* < 0.001), while after the treatment, MMR was not correlated with behaviors or RMR (Table 2). Prior to the SSRI treatment, a strong negative correlation was observed between the coping style and stress reactivity (*r* = −0.60, *P* < 0.0001), suggesting a behavioral syndrome (Fig. 5a). After the treatment with antidepressants, however, the syndrome disappeared (Table 2, Fig. 5b).

**Table 2:** Pearson correlation coefficients between traits in crickets before SSRI treatment (below diagonal) and after SSRI treatment (above diagonal); lower and upper 95% CI values are presented in parentheses; all *P*-values are Bonferroni corrected.

Traits	Body mass	Coping styles	Stress reactivity	RMR	MMR
Body mass		−0.14 (−0.34; 0.06)	−0.63^****^ (−0.73; −0.53)	0.40^***^ (0.22; 0.55)	−0.08 (−0.29; 0.13)
Coping styles	0.69^****^ (0.59; 0.78)		0.00 (−0.17; 0.17)	0.33^**^ (0.8; 0.53)	0.09 (−0.09; 0.26)
Stress reactivity	−0.66^****^ (−0.74; −0.57)	−0.60^****^ (−0.73; −0.47)		−0.32^*^ (−0.48; −0.15)	−0.03 (−0.21; 0.16)
RMR	0.63^****^ (0.50; 0.74)	0.69^****^ (0.56; 0.79)	−0.74^****^ (−0.80; −0.67)		0.09 (−0.10; 0.27)
MMR	0.34^**^ (0.13; 0.52)	0.37^**^ (0.20; 0.53)	−0.40^***^ (−0.55; −0.24)	0.40^***^ (0.21; 0.56)	

^*^P < 0.05; ^**^P < 0.01; ***P < 0.001; ^****^P < 0.0001; (all Bonferroni corrected).

## Discussion

**Figure 6:**
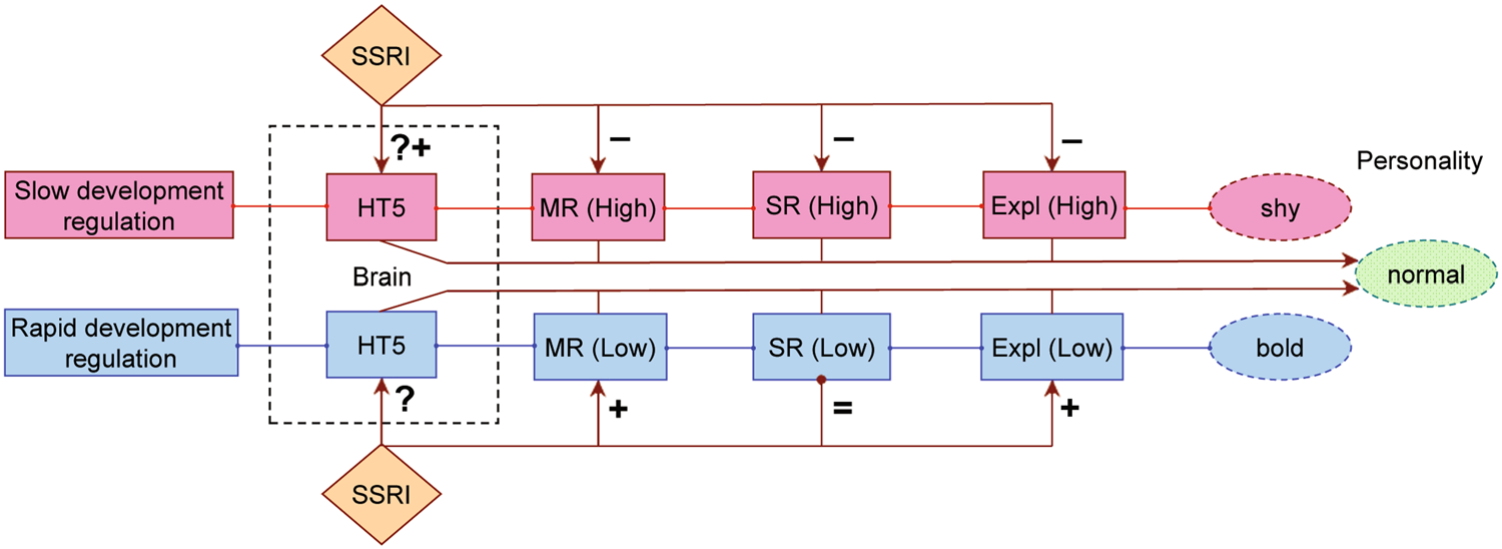
A flow chart showing trait associations and the effects of SSRI treatment in slowly and rapidly developing crickets. Slow development is associated with higher (resting and maximum) metabolic rates (MR), stress reactivity (SR), exploration (Expl) and shy personality. Rapid development is associated with lower (resting and maximum) metabolic rates, stress reactivity, exploration and bold personality. By presumably raising 5-HT levels in the brain, SSRI decreases key trait values (MR, SR, Expl) in slowly developing crickets and raises these values in rapidly developing crickets. This results in the “normal” (intermediate) personality phenotype in slowly and rapidly developing crickets that is typical of the control line.

Evidence shows that slow development is associated with upregulation of stress-related genes^53^. In humans, a predisposition toward experiencing negative emotions and low inhibitory control are linked to sickness and many psychiatric conditions^54–56^. It is likely that slower development in crickets confers higher levels of anxiety/neuroticism along the stress reactivity axis since the SSRI treatment increased the time to resume movements (*i.e*. decreased anxiety) under the stressful conditions of the insect chamber. The relaxed stress reactivity under the SSRI treatment was associated with significant decreases in the RMR and MMR of slowly developing crickets (Fig. 6). The SSRI treatment presumably increased the concentration of 5-HT in the brain, while 5-HT could have lowered body temperature by decreasing the metabolic rate (Fig. 6). 5-HT activates glycolysis, forming lactic acid. Excess lactic acid tends to decrease efficient energy production by interfering with mitochondrial respiration^57,58^. On the other hand, the SSRI treatment did not affect stress reactivity of rapidly developing crickets. Moreover, the treatment that brings higher concentration of brain 5-HT increased RMR, MMR and exploration time of rapidly developing, often bold crickets, while it decreased exploration time of slowly developing, often shy crickets. High concentration of brain 5-HT may cause a *serotonin syndrome* that often includes high body temperature, indicating a higher metabolic rate^59^.

Richmond *et al*.^60^ found that aquatic invertebrates are highly sensitive to pharmaceutical pollution in their environment: dipteran midges emerged significantly earlier and the total biomass of emerged adult insects was greater in fluoxetine- and citalopram-exposed streams when compared with controls. This suggests that SSRI presence in the environment induces selection for faster development and more intense metabolism. This could mean that (i) rapidly and slowly developing crickets have different 5-HT tolerance, 5-HT sensitivity in brain receptors or different 5-HT concentration in the brain; (ii) 5-HT affects coping styles rather than responses along the stress reactivity axis and/or (iii) serotonergic metabolism is linked to growth. Under natural conditions, proactive (active) coping may have higher survival value especially under high density of conspecifics/stronger competition than a reactive (passive) coping style^16^. On an evolutionary scale, however, survival values for different behavioral types generally depend on environmental variability and predictability^61^.

Prior research has revealed differences in sensitivity to 5-HT levels in *D. melanogaster*^62^. *Ns3* mutant flies (which reach less than 60% of normal size and have fewer and smaller cells, but exhibit normal body proportions) and wild-type flies were treated with 5-HTP (5-hydroxy-L-tryptamine), the immediate precursor of 5-HT. This treatment resulted in a developmental delay in wild-type animals, reminiscent of the *ns3* mutant phenotype, while *ns3* mutants were more sensitive to 5-HTP than their wild-type counterparts. This suggests that *ns3* mutants have already increased serotonergic signaling, as is likely in rapidly developing crickets. Using selective 5-HT1A and 5-HT1B agonists, de Boer and Koolhaas^52^ found a manyfold difference between high- and low-aggression male rats. The high-aggression rats had a far more sensitive 5-HT1A auto-receptor and 5-HT1B receptor-mediated inhibition of the serotonergic neurons than low-aggression rats. However, the mechanisms that underlie these effects remain elusive. One reason is that the spatial distribution of serotonergic receptors remains poorly located and characterized in both the vertebrate and insect brain. As pointed out by de Boer and Koolhaas^52^, it is crucial to know whether the receptors are located postsynaptically or presynaptically. At the presynaptic level, 5-HT agonists reduce 5-HT signaling, whereas at the level of the postsynaptic receptors, they mimic the effects of enhanced 5-HT signaling. In future research on 5-HT1A and 5-HT1B, agonists need to be tested in slowly and rapidly developing crickets to reveal any differences in the sensitivity of their 5-HT receptors and its relation to the development of personality along the stress reactivity and coping styles axes.

Interestingly, the SSRI treatment decreased stress reactivity (*i.e*. increased the time to resume activity) only in slowly developing crickets while it affected exploration time and coping styles of both slowly and rapidly developing individuals, but in the opposite way. Koolhaas *et al*.^29^ suggested a mechanism to explain the opposing effect of 5-HT on coping styles of individuals having to different personalities. Evidence shows that activation of the 5-HT1A auto-receptor leads (i) to a reduction of the preferred behavioral response in a forced swim test in rats and (ii) an increase in an alternative behavioral response rather than to a general reduction in anxiety^63^. It is known that 5-HT1A receptor agonists in the reactively coping males show a reduction of immobility and an increase in escape attempts, whereas the proactively coping animal shows a decrease in escape and an increase in immobility behavior in the test^64^. Koolhaas *et al*^29^. concluded that the effect of 5-HT may be related to the coping styles axis rather than to the emotionality axis. This idea is supported by experimental evidence obtained in the current study, where we show that 5-HT may be involved in regulation of behavior not only along a stress reactivity gradient but also along a coping styles axis. These findings indicate that personality types mediate the strength and direction of 5-HT treatment, which should be taken into account in research on behavior and the underlying metabolism, the influence of pharmacological compounds on behavioral responses and developing new types of antidepressants. The proximate task of future research would be to reveal the types of 5-HT receptors that are involved in cricket behavioral responses and whether different receptor subtypes are engaged in stress-induced redistribution of immune reactions during behavioral responses.

It is important to explain the link between developmental speed and body size found in this study, since it implies greater complexity in the structure and development of personality than expected before^16^. We found that slowly developing crickets were significantly larger than rapidly developing crickets, which is in line with earlier data on this species^43^. Although faster developmental time in rapidly developing individuals may explain their smaller body size, rapidly developing, often bold crickets are less anxious and can allocate more time to feeding, thus compensating for shorter time frames left for growth. The fact that the SSRI treatment affected slowly and rapidly developing crickets in the opposite way may indicate that rapidly developing crickets have higher baseline 5-HT in their brain than slowly developing crickets.

In *D. melanogaster*, neuromodulatory actions of 5-HT were shown to depress feeding, while decreased neuronal 5-HT levels increased appetite^65^. In another dipteran species, the flesh fly *Neobellieria bullata*, 5-HT injection in the hemolymph decreased feeding^66^. 5-HT and its effects on feeding are necessary to induce behavioral gregarization in migratory desert locusts^34^. The decrease in body size is among the most obvious effects of phase transition in morphological appearance from solitary to gregarious forms of locusts^67,68^. Consistent with these findings, reduction in the brain 5-HT signaling by injection of a serotonergic-specific neurotoxin induces hyperphagia and increased growth in rats^69^. This may explain the larger body size of the slowly developing, often shy crickets. The observation that *D. melanogaster ns3* mutants consume the same amount of food as control flies during early larval stages also suggests that serotonergic signaling appears to coordinate feeding behavior with growth at the organismal level^62^. Importantly, Kaplan *et al*.^62^ found close apposition of the axonal projections of serotonergic neurons to the insulin-producing cells (IPCs) in the brain of *D. melanogaster*, suggesting that these cells communicate to coordinate growth with 5-HT-modulated behaviors and environment. They showed that in *D. melanogaster*, insulin-like peptides both regulate blood sugar and act as growth factors, where serotonergic neurons control the adult body size by affecting insulin-like peptide secretion^62,70^. This calls for studies on possible similar synergies in cricket brains and bodies to define the functional patterns of the serotonergic synapse and subsequent effects on the phenotype. This may help to understand how and why 5-HT regulates somatic growth and the development of personality. For example, behaviors and sensitivity of 5-HT receptors might depend not only on the concentration of 5-HT but also on the proximity of the IPCs and serotonergic neurons in the brain.

In conclusion, we show that personality-indicating consistent behavioral responses in crickets are tightly linked to developmental speed and body size. Antidepressants affect exploration and stress reactivity in different ways: we observed a higher SSRI impact on the coping styles axis than on the stress reactivity axis. Moreover, we found the opposite type of reactions to SSRI treatment in slowly and rapidly developing crickets along the coping styles axis. On the other hand, there were no simple one-way decreases or increases in exploration time at the two ends of the bimodal distribution of developmental speed / behavioral responses. This study shows that the SSRI treatment significantly decreased the variability of behaviors along the coping styles and stress reactivity axes, thus suggesting that antidepressants reduce variation in personality-related behaviors. It is also important to explain the link between developmental speed and body size found in this study, since it suggests greater complexity in the structure and development of personality than expected before^16^. Overall, our results may have limited explanatory power with regard to the magnitude of the SSRI effects on behaviors. This is because we have not studied SSRI activity in a dose-dependent manner, nor do we have data on possible side effects of SSRI treatment on cricket behavior(s). However, the high repeatability values that we recorded on the observed behaviors along the coping styles and the stress reactivity axes have the potential to show the diversity of directions that SSRI effects can take, and how these vary based on developmental speed and personality of the animals.

Taken together, the present findings call for more research in brain neurophysiology and receptor distribution, a better definition of synaptic function, involvement of more personality axes and personality types (not only shy and bold ones), and an improved understanding of the brain concentrations of 5-HT and dose-dependent 5-HT effects in animals. Finally, this study shows that slowly and rapidly developing crickets markedly differ in behavioral traits and metabolism. Slowly developing individuals were larger, more explorative, less bold and they had higher RMR and lower MMR compared to rapidly developing crickets before the SSRI treatment. Overall, these findings do not support recent ideas concerning the pace-of-life syndrome (POLS) hypothesis^16^, which predict higher exploration and metabolic rates in rapidly developing bold animals.

## Methods

### Insects and selection lines

We studied crickets from a laboratory stock originating from a wild population (Davis, California, USA). The animals were selected for developmental speed; crickets of the 5^th^ generation were investigated in this study. To start the selection lines, a total of 903 juvenile wild crickets were used. Until the crickets reached maturity, they were kept individually in plastic containers (280 mm × 115 mm × 105 mm) with a ventilation hole (60 mm dia.) covered with a plastic net. They were kept in a 12:12 h light–dark cycle and 26 ± 1 °C, with food and water *ad libitum* before and during experimental trials. The animals were selected for rapid and slow development, and we also maintained control groups. For rapid and slow development lines, mated males and females were selected according to their maturation age, with maximum 16.67% of the most rapidly or slowly maturing individuals being used, while in the control line the matings were randomized over the whole maturation age range^28^. The first three generations were reared at the University of Eastern Finland, while the rest of the rearing of the lines occurred in Estonia.

After five generations of selection for developmental speed, the development time (the average maturation time ± SD) for rapidly developing individuals was 98.03 ± 11.72 days (n = 238 crickets), 120.28 ± 25.12 days (n = 284) for the control individuals and 138.41 ± 23.05 days (n = 269) for slowly developing crickets. The mean longevity after eclosion for all three groups was 88.50 ± 23.54 days (one-way ANOVA, *F*_(2, 788)_ = 25.79, *P* < 0.001). Longevity of males and females of all lines did not differ significantly (one-way ANOVA, *F*_(1, 789)_ = 1.0, *P* = 0.318). Lifespan after eclosion of rapidly developing crickets (83.95 ± 23.52 days) and the control individuals (84.93 ± 22.06 days) did not differ significantly (Tukey’s test, *P* > 0.05), while lifespan after eclosion of slowly developing crickets (96.61 ± 25.05 days) significantly differed from the mean longevities of rapidly developing and control crickets (Tukey’s tests, both *P* < 0.001).

### Trials, measurements and antidepressants

We tested whether escitalopram, a selective serotonin reuptake inhibitor (SSRI) (Brand name: CIPRALEX, CFL Pharmaceuticals Ltd) has any effect on cricket behavior and metabolism. Escitalopram is the (*S*)-stereoisomer (Left-enantiomer) of the citalopram prescribed frequently to humans, low concentrations of which have been detected in natural habitats^60,71–74^. Before the SSRI was added to the food of crickets, coping style (exploration) tests (day 1), and stress reactivity tests, measurements of RMR and MMR (day 2) were carried out for each of the F5 generation crickets (slow, n = 37; rapid, n = 41; control, n = 28) 15 days after maturation at the imago stage. To assess repeatability, we carried out coping style tests, stress reactivity tests and measurements of RMR and MMR one more time on days 3 and 4. As soon as these trials were done, on day 5 we started adding the SSRI to the food of the crickets (0.5 mg/kg per day added to Tetra Fish Food Flakes: Tetra, Blacksburg, VA) for twelve days (days 5–16). On days 13 and 14, the behavioral tests (coping styles and stress reactivity) and metabolism measurements were done again using the same animals as used during the previous trials. To assess repeatability of the behavioral variables, we repeated the tests on days 15 and 16.

The insects were weighed before each measurement of metabolic rate using a Kern analytical balance (Kern & Sohn GmbH, Balingen, Germany). RMR and MMR values were corrected for body mass^28^. Behavioral trials were conducted under constant temperature (24 ± 1.5 °C) and soundproof conditions. We used dim red light (25 W red incandescent bulb) since *Gryllus* spp. cannot see long (red) wavelengths properly^75^, which allowed us to observe the nocturnal insects without disturbing them. The crickets were provided with drinking water before the onset of the trials, while food was removed 3 h prior the beginning of experimental trials. All behavioral trials were performed between 17:00 and 24:00 h. The observers of insects’ behavior and metabolic responses were blind to all predictions and treatment^76^.

### Tests under familiar non-stressful environment: coping styles (exploration)

The shelter part and the food area were separated by a corridor within individual containers. The crickets were trained for a week to run through a corridor (3 cm wide and 10 cm long) to reach the food after a 3 h fast. During the fast, water was provided *ad libitum*. We used a one-cent Euro coin as a novel object before the crickets received the SSRI, while a one-dime USD coin was used during the SSRI treatment. The novel objects were placed on the floor of the corridor 5 cm away from the entrance. We randomly chose the side of the corridor in which to place a novel object, and we always placed it on the opposite side during the second trial. As soon as the door between the shelter and the corridor was opened and the animal started to move through the corridor, we recorded the time it took to reach the food area. Since the crickets did not make any stops in the corridor when a novel object was not present, the time spent in the corridor reflects exploration activity / coping style of each individual – the extent of paying attention to changes in the environment.

### Tests under unfamiliar environments: stress reactivity

Next day after the coping style test, we handled each cricket for 1 min and then gave it an opportunity to escape into a conical plastic Eppendorf test tube (volume 5 ml) kept in dark conditions, which was used as an insect chamber during the measurement of RMR. This insect chamber represented a novel and stressful environment to the crickets. Resuming activity thus reflected each individual’s level of anxiety in this stressful environment. We assumed that the higher the anxiety, the sooner the individual will resume active body movements to avoid the stressful conditions of the windy and noisy insect chamber^26,27^. The latency to resume activity indicates the duration of freezing or immobile state, a widespread anti-predator response used by many taxa^77,78^. The insect chamber was connected to the respirometer by means of rubber tubing^26^. Upon escape to the insect chamber, each of the crickets immediately became completely immobile, as if hiding in a burrow or a crevice. We waited until all crickets resumed active movements, recorded the time between entering the tube and resuming activity, and used it as a measure of stress reactivity.

### Measuring RMR and MMR

The insects remained in their metabolic chambers until their CO_2_ emissions (and therefore their RMRs) reached the lowest stable rates. We also measured MMR at times when the crickets’ movements reached the highest rates^79^ while they performed struggling movements. As soon as the measurements were over, we returned the crickets back to their plastic housing-boxes.

The LI-7000 differential CO_2_/H_2_O analyzer (LiCor, Lincoln, NE, USA) was calibrated by means of calibration gases with gas injection^12,13,26^. While measuring CO_2_ emissions, the insect chamber was perfused with dry CO_2_-free air at a flow rate of 60 ml min^**−**1^. The respirometric device was combined with an infrared optical system using IR-emitting and IR-sensor diodes^12,13^. IR-diodes made it possible to record CO_2_ production and to follow the movement intensity of each cricket.

### Statistical analyses

We used repeated measures ANOVAs (rmANOVAs) to assess changes in body mass, coping style, stress reactivity, resting metabolic rate and maximum metabolic rate after antidepressant treatment (SSRI, within-subjects factor) in the same subjects with developmental line and sex used as between-subjects factors. We reported only the main effects when no significant interactions between between-subjects factors and/or interactions between within-subjects factor (antidepressant treatment) and between-subjects factors were found; otherwise the simple main effects using post hoc Bonferroni test were also reported. In order to account for age and testing apparatus effects, we performed rmANOVA with untreated (no antidepressant was given) groups of crickets (slow, n = 20; rapid, n = 20; control, n = 20). Analyses were performed in Statistica 8.0 for Windows (StatSoft Inc., Tulsa, OK, USA). To assess repeatability, we calculated the intraclass correlation coefficient using a two-way random effects model for single measures^80^. Pearson correlations were used to assess associations between different traits. Lower and upper 95% CI limits of correlation coefficient were calculated using non-stratified bootstrapping sampling with 2000 iterations.
